# Molecular Targets Related Drug Resistance Mechanisms in MDR-, XDR-, and TDR-*Mycobacterium tuberculosis* Strains

**DOI:** 10.3389/fcimb.2018.00114

**Published:** 2018-04-10

**Authors:** H. M. Adnan Hameed, Md Mahmudul Islam, Chiranjibi Chhotaray, Changwei Wang, Yang Liu, Yaoju Tan, Xinjie Li, Shouyong Tan, Vincent Delorme, Wing W. Yew, Jianxiong Liu, Tianyu Zhang

**Affiliations:** ^1^State Key Laboratory of Respiratory Disease, Guangzhou Regenerative Medicine and Health Guangdong Laboratory, Guangzhou Institutes of Biomedicine and Health, Chinese Academy of Sciences, Guangzhou, China; ^2^University of Chinese Academy of Sciences, Beijing, China; ^3^Institute of Health Sciences, Anhui University, Hefei, China; ^4^State Key Laboratory of Respiratory Disease, Guangzhou Chest Hospital, Guangzhou, China; ^5^Tuberculosis Research Laboratory, Institut Pasteur Korea, Seongnam-si, South Korea; ^6^Stanley Ho Centre for Emerging Infectious Diseases, The Chinese University of Hong Kong, Hong Kong, China

**Keywords:** *Mycobacterium tuberculosis*, drug resistance, molecular, comorbidities, therapeutic, drug targets

## Abstract

Tuberculosis (TB) is a formidable infectious disease that remains a major cause of death worldwide today. Escalating application of genomic techniques has expedited the identification of increasing number of mutations associated with drug resistance in *Mycobacterium tuberculosis*. Unfortunately the prevalence of bacillary resistance becomes alarming in many parts of the world, with the daunting scenarios of multidrug-resistant tuberculosis (MDR-TB), extensively drug-resistant tuberculosis (XDR-TB) and total drug-resistant tuberculosis (TDR-TB), due to number of resistance pathways, alongside some apparently obscure ones. Recent advances in the understanding of the molecular/ genetic basis of drug targets and drug resistance mechanisms have been steadily made. Intriguing findings through whole genome sequencing and other molecular approaches facilitate the further understanding of biology and pathology of *M. tuberculosis* for the development of new therapeutics to meet the immense challenge of global health.

## Introduction

Tuberculosis (TB) is an airborne infectious disease caused by *Mycobacterium tuberculosis* (Arnold, 2007; Lillebaek et al., 2016). During the eighteenth and nineteenth centuries, this disease widely plagued Europe and North America and was known as “Captain among these men of Death.” In the 1800's, a person with TB was advised to “take slumber and ingest nutritious diet,” due to lack of knowledge about this disease and the absence of medication (Keshavjee and Farmer, 2012). Later, the discoveries of tuberculin in 1890, Bacille-Calmette-Guérin (BCG) vaccine in 1908, streptomycin (STR) in 1944 and isoniazid (INH) in 1952 (Daniel, 2006) were all perceived as scientific revolutions that could finally lead to the eradication of this deadly disease (Fogel, 2015). The emergence of drug-resistance as well as infection and transmission of the human-immunodeficiency virus (HIV) further demonstrated the need of better weapons to eliminate TB. Soon after, researchers realized that more advanced investigations would be required for development of rapid diagnostic tools and effective chemotherapy to reduce drug resistant-TB morbidity, mortality and risk of transmission.

According to the World Health Organization (WHO), 10.4 million TB cases were reported in 2016, including 1 million people co-infected with HIV, 1.9 million were attributable to malnutrition, 0.8 million to diabetes and 0.8 million to smoking. The same year, TB caused 1.3 million deaths among HIV-negative patients and additionally 374,000 deaths among HIV-positive patients (WHO, 2017). *M. tuberculosis*, unlike other pathogens, requires an extended anti-TB therapy treatment of at least 6 months in order to achieve complete sterilization and prevent relapse. Chemotherapy of TB is required to tackle growing and semi-dormant/dormant bacilli, as well as to restrain the emergence of drug resistance. Unfortunately, development of drug resistance in the form of monoresistant-TB or MDR-/XDR-/TDR-TB critically obstructs the efficacy of currently available drug regimens. Improper use of antibiotics, lack of treatment adherence, mutational modifications, and limited access to drugs or diagnostic tools all are risk factors associated with the emergence of resistant forms of TB (Lange et al., 2014; Georghiou et al., 2017; Manson et al., 2017). Besides, acquired drug-resistant TB and transmitted drug-resistant TB pose a serious threat to global TB control and population health. To meet the evolving challenge of TB today, newly developed anti-TB drugs and vaccines are urgently needed.

Furthermore, several studies strongly emphasize that pathoadaptive mutations and the mutations in drug-related genes (e.g., genes responsible for drug activation/conversion) are mainly responsible for numerous pathways in the evolution of pathogenic organisms and emergence of drug-resistant strains, respectively (Day et al., 2001). In this review we mainly focused on molecular targets containing mutations which are found to be involved in development of drug resistance in *M. tuberculosis*.

## MDR-, XDR-, and TDR-TB and their global extent

WHO identified that TB is among the top 10 leading causes of death around the globe and caused more deaths than HIV in 2015 and 2016. Monoresistance to STR in *M. tuberculosis* was first reported in 1947-48 (Crofton and Mitchison, 1948). To overcome drug resistance development, initially, combined therapy including INH and para-aminosalicylic acid (PAS) along with STR, and subsequently addition of pyrazinamide (PZA) and rifampicin (RIF) had been practiced. However due to poor physician prescription, and/or poor patient adherence, and/or poor drug quality/supply, and factors not commonly encountered or well known, drug resistance can be acquired and escalated to involve both RIF and INH–MDR-TB (Frieden et al., 1993). In 2006, Centres for Disease Control and Prevention (CDC) and WHO jointly reported XDR-TB (MDR-TB with additional bacillary resistance to any fluoroquinolone (FQ) and at least one of the three second-line injectable drugs [i.e., amikacin (AMK), kanamycin (KAN), and capreomycin (CAP); Gandhi et al., 2006]. In the recent past, XDR strains of *M. tuberculosis* when tested to be resistant against rifabutin (RFB), clofazimine (CLO), dapsone, clarithromycin (CLR), and thiacetazone (THZ) alongside conventional first-line drugs (FLD) and second-line drugs (SLD), were proposed as “XXDR-TB” in Italy (Migliori et al., 2007). The term “TDR-TB” was proposed by Iranian researchers to describe the “XXDR-TB” (Velayati et al., 2009). Later, TDR-TB was also reported in India (Udwadia et al., 2012) and South Africa (Klopper et al., 2013).

In addition, two new drugs bedaquiline (BDQ) and delamanid (DMD) were also found to be ineffective against TDR-TB (Maeurer et al., 2014). Moreover, molecular analysis identified the mutations in the genomic sequence are the core cause of drug-resistant TB. MDR and XDR-TB are extremely serious disease worldwide as these can progress to TDR-TB with seemingly higher mortality than cancer by 2050 (Roca et al., 2015; Furin et al., 2016). WHO report of 2016 publicized the global extent of TB/HIV, MDR-, XDR-, and TDR-TB (Table 1). The collected data suggests the burden of TB in India is significantly higher than previously estimated, and slightly rising trends for the North Korea and the Philippines were also noticed. Moreover, India, China and the Russian Federation accounted altogether for 45% of the total RIF-resistant/MDR-TB burden. By the end of 2015, a total of 7579 XDR-TB cases were reported from 74 countries, which were more than twice the sum of 2014 report (WHO, 2016). The CDC stated in 2013 that XDR-TB patients have been identified in most of the regions of the world, including the United States. Interestingly, Australia was comparatively protected from DR-TB because of its strict public health policies as nearly 90% of TB cases occurred in the immigrant communities (Toms et al., 2015).

**Table 1:** Global Extent of MDR-, XDR-, and TDR-TB (WHO, 2016).

**New TB cases in 2015 10.4 million**	**TB Deaths in 2015 1.4 million**	**Countries with 60% of the new TB cases**	**Countries with high burden of TB, MDR-TB and TB/HIV**	**Highly reported XDR-TB cases among 74 countries**	**Global HIV-Positive TB cases 500,564**	**Global MDR/RR-TB cases 132,120**	**Global XDR-TB cases 7,579**	**TDR-TB reported countries**
5.9 million in (56%) men	62% deaths in men	India	Angola, China, The Democratic Republic of the Congo, Ethiopia, India, Indonesia, Kenya, Mozambique, Myanmar, Nigeria, Papua New Guinea, South Africa, Thailand, Zimbabwe	India (2,130)	Africa = 380,032	Africa = 26,929	Africa = 1100	Italy (Migliori et al., 2007) Iran (Velayati et al., 2009) India (Udwadia et al., 2012) South Africa (Klopper et al., 2013)
3.5 million (34%) in women	25% in women	Indonesia		Ukraine (1,206)	The Americas = 21,885	The Americas = 4,489	The Americas = 122	
1 million (10%) in children	13% in children	China		The Russian Federation (1,205)	Eastern Mediteranean = 1,456	Eastern Mediteranean = 4,081	Eastern Mediteranean = 117	
Co-infected with HIV 1.2 million (11%)	Co-infected with HIV 0.4 million deaths	Nigeria		South Africa (719)	Europe = 16,137	Europe = 42,646	Europe = 2,691	
149, 000 cases of zoonotic TB	250,000 deaths from MDR/RR-TB	Pakistan		Total = 7,579 XDR-TB cases in 74 countries	South-East Asia = 64,238	South-East Asia = 35,953	South-East Asia = 3,099	
480,000 MDR-TB		South Africa			Western Pacific = 16,816	Western Pacific = 18,022	Western Pacific = 450	
100,000 RR^a^-TB								
7,579 XDR-TB								

a *RR, RIF-resistant*.

## Clinical diagnosis of DR-TB

*M. tuberculosis* is a slow growing mycobacterium, which results in prolonged duration of drug susceptibility test (DST) on solid media from 4 to 6 weeks whereas in liquid media from 1 to 2 weeks. The lack of rapid diagnostic tools can be proposed as a risk factor for the prevalence of MDR-, XDR-, and TDR-TB, because the use of inefficient drugs during the initial treatment phase may promote the appearance of drug-resistant profiles. In this context, the WHO recommend the rapid and more advanced *in vitro* growth monitoring system, MGIT 960, as well as nucleic acid amplification method, Xpert MTB/RIF for early diagnosis of PZA-resistant and RIF-resistant TB, respectively (Lawn et al., 2013). Other devices using line probe assay (LPA) technology are now available, like Genotype MTBDRplus for rapid analysis of MDR-TB (Jacobson et al., 2013) and its advanced version Genotype MTBDRsl (Barnard et al., 2012) to diagnose XDR-TB.

New approaches have also been reported, like FASTPlaque-response bacteriophage assay (Minion and Pai, 2010), microscopic observation drug susceptibility assay (MODS) (Peter et al., 2016), use of dyes to monitor bacterial growth (Coban et al., 2016), sequencing and hybridization, reverse hybridization, direct sequencing (Deggim-Messmer et al., 2016), TB Biochip platform (Xue et al., 2016) and other molecular approaches. Each method was proposed to provide faster as well as more reliable results for early detection of drug-resistance. An important consideration is the cost and the infrastructure requirements of each technique, as this is the key factor that will ultimately limit their clinical use in many regions around the world.

## Resistance causing mechanisms

Drug-resistant TB is mainly associated with chromosomal mutation mechanisms, in particular single nucleotide polymorphisms (SNP). The resistance causing factors that affect the mutation rate can be divided mainly into two groups; (i) cellular mechanisms, for example, inefficiency of mismatch repair, microsatellites, inadequate translations and error-prone DNA polymerases and (ii) external stress factors, including absence of rapid diagnostic facilities, improper anti-TB drugs prescribing practices, host environment and exposure to smoking or pollution (McGrath et al., 2014). Additionally, clinical outcomes of antibiotic combinations may also be influenced by inter-individual heterogeneities in drug pharmacokinetic and pharmacodynamics (Srivastava et al., 2011). Poor adherence of TB patients and costs required to achieve complete cure are probably the two main factors responsible for emergence of drug-resistance.

Indeed, most of the MDR-/XDR-TB patients had been treated previously either with ineffective anti-TB drugs or for suboptimal duration, resulting in an incomplete sterilization of the lungs and re-growth of “persisters” that became more resistant to the drugs which had been formerly practiced and turned them into XDR-/TDR-TB, respectively (Dalton et al., 2012). According to another study, development of drug resistance is mainly because of spontaneous mutations in drug targets which ensure the survival of TB bacilli at very low pH through induction of acid resistance, drugs adaptations, inactivity or degradation of drugs through enzymes encoded by genes of *M. tuberculosis*, molecular simulation of drug targets, and epigenetic drug tolerance (Jenkins et al., 2009; Smith et al., 2013).

Some previous anti-TB drugs are also found to be ineffective in preventing mycolic acid synthesis in DR-TB bacilli due to mutations in drug targets (Telenti et al., 1993; Ramaswamy and Musser, 1998; Gillespie, 2002; Watanabe et al., 2002). Therefore, other new targets should also be considered to control the pathways of drug resistance by new and more effective antituberculars. However, two new drugs, DMD and pretomanid may have better action on cell wall synthesis (Stover et al., 2000; Gler et al., 2012). Besides this, the role of efflux-pumps in drug-resistance in TB should not be underestimated, as reported for INH (Machado et al., 2012) and indicated by recent reports involving the membrane transporter MmpL5 in resistance to BDQ (Hartkoorn et al., 2014). Finally, concomitant bacterial infections and lack of new pharmacophore could be additional causes for the rapid emergence of MDR/XDR and TDR-TB (Srivastava et al., 2011; Vadwai et al., 2011; Machado et al., 2012; Dharmadhikari et al., 2013; Grossman et al., 2014; Lange et al., 2014). It is also worth noticing that the term “resistance” should be used carefully because misconception or misapplication of this may devise an idea that resistance is a binary phenotype, whereas it can be evaluated at multiple levels, i.e., low, moderate and high-level drug resistance (Böttger, 2011).

## DR-TB and comorbidities

Several factors are associated with a worsening of TB infection. These include HIV (Das and Horton, 2010), diabetes mellitus (Jeon and Murray, 2008), cancer (Vento and Lanzafame, 2011), solid organ transplantation (Skrahina et al., 2012), renal disease (Wu et al., 2013) tumor necrosis factor alpha (TNF-α) antagonist treatment (Kisacik et al., 2016), alcohol abuse (Stoffels et al., 2013), tobacco use (Glickman and Schluger, 2016), air pollution, malignancies and an aging population (Negin et al., 2015). The development of TB disease is estimated to be 26- to 31-fold higher in people living with HIV than those without HIV infection in 2015 (WHO, 2016). In 2015, more than 28% of TB deaths were HIV positive, which describes a strong link between the two infections (HIV and *M. tuberculosis*).

A major feature of HIV infection is the chronic T-cell activation and progressive loss of CD4^+^ T-lymphocytes, which predispose the host to active TB. Depletion of CD4^+^ T-cells in HIV subjects with latent TB infection disrupts the steadiness and structure of TB granulomas in the lung, thereby promoting the progression of infection to disease by 20-fold (Geldmacher et al., 2012). Recently, the emerging facts indicated that clinical findings including lower lung field lesions, cavities, and acid-fast bacilli (AFB) smear positivity were at higher frequencies among TB patients having comorbidity with diabetes mellitus and suffered increased risk of treatment failure, relapse, and death (Carreira et al., 2012; Workneh et al., 2016). The WHO report signifies both TB and HIV infections were influenced by the increased burden of diabetes mellitus in Sub-Saharan African countries (WHO, 2016).

## Molecular targets related drug resistance mechanisms in MDR-/XDR-/TDR-TB

**Figure 1:**
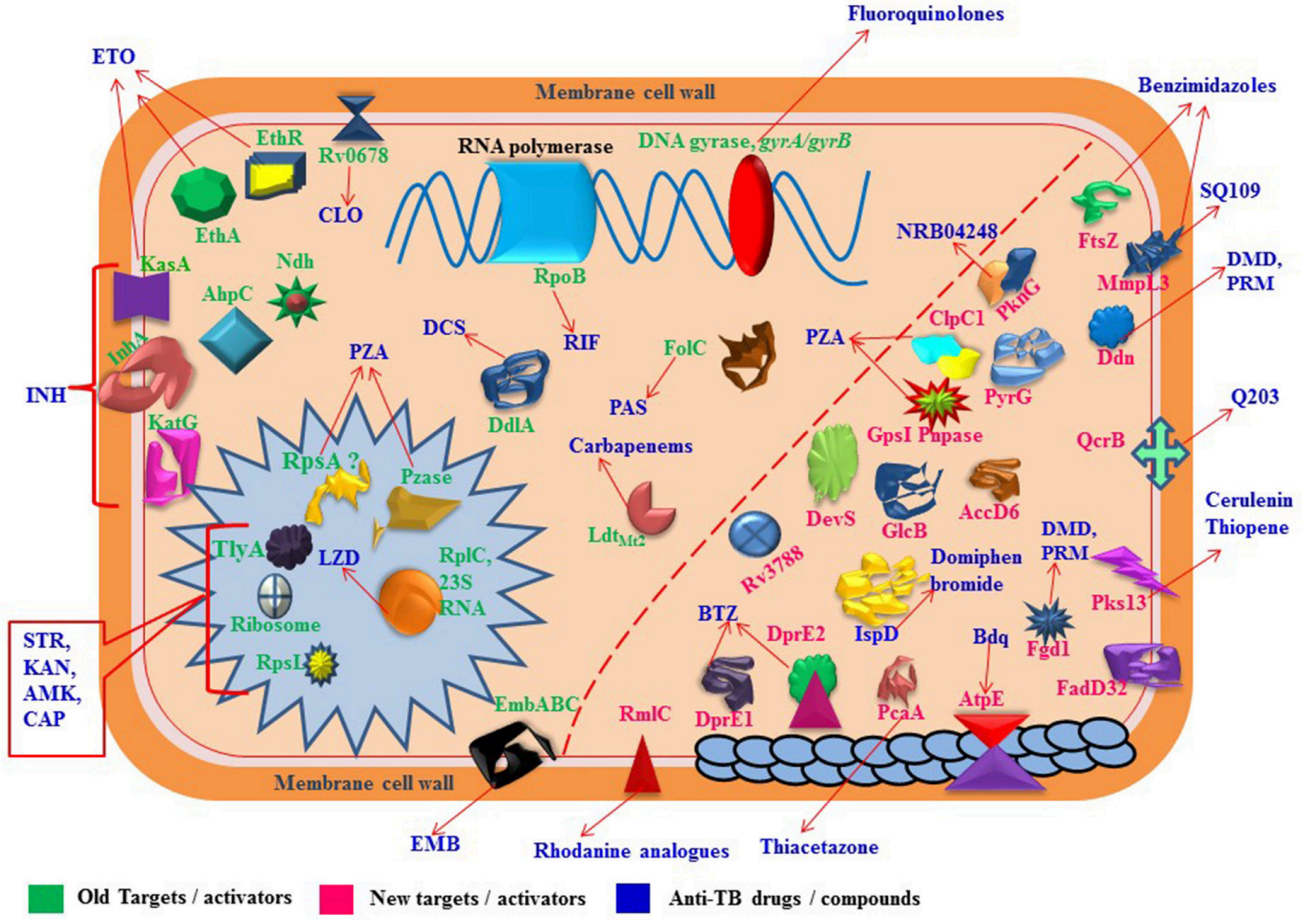
Proteins/RNAs/genes related to anti-TB drugs. 2'-O-methyltransferase TlyA (*tlyA*), Ribosomal RNA 16S (*rrs*), 30S ribosomal protein S12 RpsL (*rpsL*), 50S ribosomal protein L3 RplC (*rplC*), arabinosyltransferases EmbABC (*embABC*), DNA topoisomerase II /DNA gyrases GyrA, GyrB (*gyrA, gyrB*), RNA polymerase β-subunit RpoB (*rpoB*), catalase-peroxidase KatG (*katG*), alkyl hydroperoxidase C AhpC (*ahpC*), NADH dehydrogenase Ndh (*ndh*), enoyl-acyl-carrier protein-reductase InhA (*inhA*), β-ketoacyl ACP synthase KasA (*kasA*), Monooxygenase EthA (*ethA*), Transcriptional regulatory repressor protein EthR (*ethR*), Pyrazinamidase/nicotinamidase PZase PncA (*pncA*), 30S ribosomal protein S1 RpsA (*rpsA*), GpsI (polynucleotide phosphorylase, *gpsI*) and Pnpase (*Rv2783c*), ClpC1 (ATP-dependent protease ATP-binding subunit, *clpC1*), Conserved protein (*Rv0678, Rv0678*), folate synthase FolC (*folC*), nonclassical transpeptidase (*LdtMt2*), D-alanyl-D-alanine ligase DdlA (*ddlA*), transmembrane transport protein MmpL3 (*mmpL3*), Cell division protein FtsZ (*ftsZ*), dTDP-4-dehydrorhamnose 3,5-epimerase RmlC (rmlC), Acetyl/propionyl-CoA carboxylase (β-subunit) AccD6 (*accD6*), Deazaflavin-dependent nitroreductase Ddn (*ddn*), Mycolic acid synthase PcaA (cyclopropane synthase) (*pcaA*), CTP synthetase for Pyrimidine biosynthesis PyrG (*pyrG*), Serine/threonine-protein kinase PknG (*pknG*), Two component sensor histidine kinase DevS (*devS*), Malate synthase GlcB (*glcB*), ATP synthase C chain AtpE (*atpE*), MEP cytidylyltransferase IspD (*ispD*), Polyketide synthase Pks13 (*pks13*), Fatty-acid-AMP ligase FadD32 (fatty-acid-AMP synthetase) (*fadD32*), Hypothetical protein (Rv3788), ubiquinol-cytochrome C reductase QcrB (*qcrB*).

Whole-genome sequencing markedly increase the detection capability to find out the mutations in molecular targets which play significant role in development of resistance against anti-TB drugs and lead toward MDR/XDR/TDR-TB (Figure 1). Concurrently, it also facilitates in rapid and precise identification of virulence factors of pathogen and can be used to explore the pathways of disease transmission (Gilchrist et al., 2015). Though, antimicrobial resistance and bacterial virulence have been considered as different aspects but there is a composite relationship between antibiotic resistance and virulence as they share few common characteristics. A detailed study by Beceiro et al. (2013) has been published to explain how bacterial virulence and fitness can be affected by drug resistance and the relationship between resistance and virulence can be influenced by different genetic mechanisms (e.g., co-selection and compensatory mutations). Several observational studies have verified that emergence of drug resistance and increased virulence often arise almost simultaneously; but, their genetic association has been relatively ignored (Schroeder et al., 2017). However, uncovering the complexities of genetic modifications and drug resistance mechanisms may recognize the new drug targets which ultimately provides the more opportunities for discovery and development of new anti-TB drugs (Vincent et al., 2012). This review mainly considers the molecular targets and mutations involved in altering the metabolic activities to develop drug resistance which directs toward severe resistant strains of *M. tuberculosis*.

*M. tuberculosis* H37Rv has a genome comprising 4,411,532 bp with high GC contents (65.9 %) containing around 4,000 protein-coding genes as well as 13 pseudogenes, 45 tRNA genes, 3 rRNA genes, 30 ncRNA genes, and 2 miscRNA genes, indicating a high protein coding percentage (91.2%) as gene density is measured 0.91 genes per Kb, so the average length is 1,002 bases per gene (Data collected from, http://genolist.pasteur.fr/TubercuList). Mutations are very important in determining the transmissibility of specific genotypes (de Vos et al., 2013). Molecular and epidemiological data assists to evaluate the transmission rate of resistance genotypes (Dye et al., 2002; Cohen et al., 2003) which actually report that fitness rate may be affected by epistasis, the phenotypic effect of a mutation depends on the presence or absence of other mutations in the same genome (Borrell and Gagneux, 2011). In fact, drug resistance based on genetic mutations is more challenging which leads toward unknown resistance mechanisms (Zhang et al., 2013). A summary of different molecular targets and the related drugs is presented in Table 2. Each target is briefly described below.

**Table 2:** Drug targets (genes/proteins), their products and functional activities and mode of action of anti-TB drugs.

**Drug targets**	**Size (bp)**	**Protein length**	**Molecular mass (Da)**	**Product**	**Functional activity**	**Mechanisms of anti-TB drugs**	**Structure & Pubchem CID**	**References**
*katG* (*Rv1908c*)	2,223	740	80,572.8	Catalase-peroxidase	Intracellular survival of mycobacteria	**INH**: Inhibition of mycolic acid synthesis **Activity**: Bactericidal	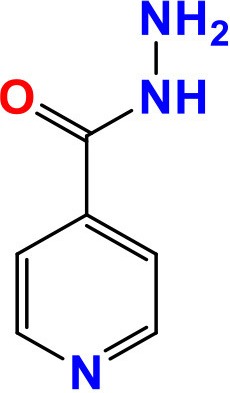 Pubchem CID: 3767	Ramaswamy et al., 2003; Vilchèze et al., 2006, 2007; Forrellad et al., 2013; Machado et al., 2013; Bantubani et al., 2014; Seifert et al., 2015; Zhang and Yew, 2015; Aye et al., 2016; Datta et al., 2016
InhA (Rv1484)	810	269	28,527.8	NADH-dependent enoyl ACP reductase	Mycolic acid biosynthesis			
AhpC (Rv2428)	588	195	21,566.3	Alkyl hydroperoxide reductase	Protection against oxidative stress			
FabG1 (Rv1483)	744	247	25,665.2	3-Oxoacyl (acyl-carrier protein) reductases	Fatty acid biosynthesis pathway			
*iniA* (Rv0342)	1,923	640	70,082.9	INH inductible gene protein IniA	Efflux pump associated			
FadE24 (Rv3139)	1,407	468	49,646.4	acyl-CoA dehydrogenase FadE24	Involved in lipid degradation and fatty acid β –oxidation			
KasA (Rv2245)	1,251	416	43,284.1	β -Ketoacyl acyl carrier protein synthase	Involved in fatty acid biosynthesis			
*ndh* (*Rv1854c*)	1,392	463	49,619	NADH dehydrogenase Ndh	Electrons transference from NADH to the respiratory chain			
*rpoB* (Rv0667)	3,519	1172	129,218	β-subunit of RNA polymerase	Catalyzes the transcription of DNA into RNA	**RIF**: Inhibition of RNA synthesis **Activity**: Bactericidal	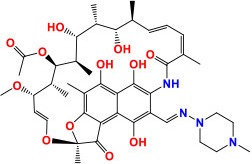 Pubchem CID: 5458213	Mboowa et al., 2014; Ocheretina et al., 2014; Thirumurugan et al., 2015; Aye et al., 2016
*rpoA* (*Rv3457c*)	1,044	347	37,706.5	α-subunit of RNA polymerase	Catalyzes the transcription of DNA into RNA			
*rpoC* (Rv0668)	3,951	1316	146,737	β′-subunit of RNA polymerase	Catalyzes the transcription of DNA into RNA			
*pncA* (*Rv2043c*)	561	186	19,604.6	Pyrazinamidase/nicotinamidase (PZase)	Converts amides into acid (PZA into POA)	**PZA**: Inhibition of trans-translation and pantothenate and CoA synthesis; depletes of membrane energy **Activity**: Bactericidal / bacteriostatic	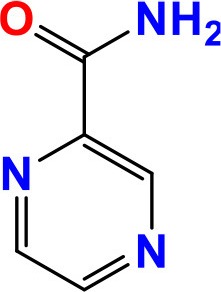 Pubchem CID: 1046	Juréen et al., 2008; Shi et al., 2011b, 2014; Njire et al., 2016, 2017; Zhang et al., 2017
RpsA (Rv1630)	1,446	481	53,201.8	30S ribosomal protein S1	Translate mRNA with a shine-dalgarno (SD) purine-rich sequence			
*panD* (*Rv3601c*)	420	139	14,885	Aspartate alpha-decarboxylase	Pantothenate biosynthesis			
*clpC1* (*Rv3596c*)	2,547	848	93,552.4	ATP-dependent protease ATP-binding subunit ClpC1	Protein degradation, Hydrolyses proteins in presence of ATP			
*gpsI* (*Rv2783c*)	2,259	752	79,734.7	Bifunctional protein polyribonucleotide nucleotidyltransferase GpsI (pnpase)	Involved in mRNA degradation			
*rpsL* (Rv0682)	375	124	13,849.2	30S ribosomal protein S12 RpsL	Translation initiation step	**STR**: Inhibition of protein synthesis **Activity**: Bactericidal	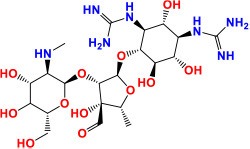 Pubchem CID: 19649	Finken et al., 1993; Sharma et al., 2007; Chakraborty et al., 2013; Du et al., 2013; Perdigão et al., 2014; Smittipat et al., 2016
*rrs* (MTB000019)	1,537	—	—	16S ribosomal RNA	Stable RNAs			
*gidB* (*Rv3919c*)	675	224	24,031.9	7-methylguanosine methyltransferase	Probable glucose-inhibited division protein B Gid			
EmbB (Rv3795)	3,297	1098	118,021	Arabinosyl transferase EmbB	Biosynthesis of the mycobacterial cell wa	**EMB**: Inhibition of arabinogalactan synthesis **Activity**: Bacteriostatic	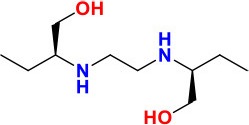 Pubchem CID: 14052	Mikusová et al., 1995; Wang et al., 2010; Safi et al., 2013; Yoon et al., 2013; Moure et al., 2014; Zhang et al., 2014; Brossier et al., 2015; Xu et al., 2015
EmbA (Rv3794)	3,285	1094	115,692	Arabinosyl transferase EmbA	Biosynthesis of the mycobacterial cell wall			
EmbC (Rv3793)	3,285	1094	117,490	Arabinosyl transferase EmbC	Biosynthesis of the mycobacterial cell wall			
*embR* (*Rv1267c*)	1,167	388	41,933.5	Transcriptional regulatory protein EmbR	Regulator of embCAB operon transcription			
*rmlD* (*Rv3266c*)	915	304	32,044.8	dTDP-4-dehydrorhamnose reductase	Involved in dTDP-L-rhamnose biosynthesis			
*gyrA* (Rv0006)	2,517	838	92,274.3	DNA gyrase subunit A	Negatively supercoils closed circular double-stranded DNA	**Fluoroquinolone**: Inhibition of DNA synthesis **Activity**: Bactericidal	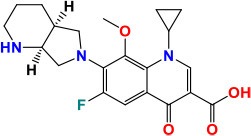 Moxifloxacin Pubchem CID: 152946	Aubry et al., 2004; Von Groll et al., 2009; Cui et al., 2011; Nosova et al., 2013; Li et al., 2014
*gyrB* (Rv0005)	2,142	675	74,058.7	DNA gyrase subunit B	Negatively supercoils closed circular double-stranded DNA			
*rrs* (MTB000019)	1,537	—	—	16S ribosomal RNA	Stable RNAs	**KAN/AMK/CAP/VIM:** Inhibition of protein synthesis **Activity**: Bactericidal	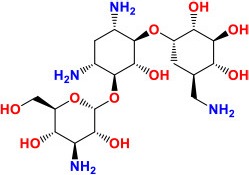 Kanamycin Pubchem CID: 6032 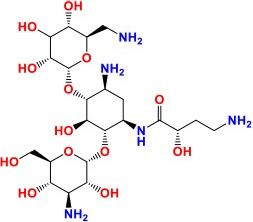 Amikacin Pubchem CID: 37768 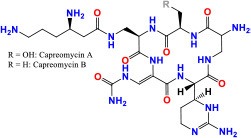 Capreomycin Pubchem CID: 3000502 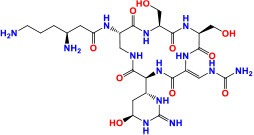 Viomycin Pubchem CID: 3037981	Stanley et al., 2010; Ajbani et al., 2011; Yuan et al., 2012; Du et al., 2013; Reeves et al., 2013; Sowajassatakul et al., 2014; Freihofer et al., 2016
*eis* (*Rv2416c*)	1,209	402	43,771.8	Aminoglycoside N-acetyltransferase	Acetylation, intracellular survival			
*whiB7* (Rv3197A)	279	92	10,106.7	Transcriptional regulatory protein WhiB7	Transcriptional mechanism			
TlyA (Rv1694)	807	268	28,042.1	2′-O-methyltransferase	Methylates 16S and 23S rRNA			
InhA (Rv1484)	810	269	28,527.8	NADH-dependent enoyl ACP reductase	Mycolic acid biosynthesi	**ETO**: Disrupts cell wall biosynthesis **Activity**: Bacteriostatic	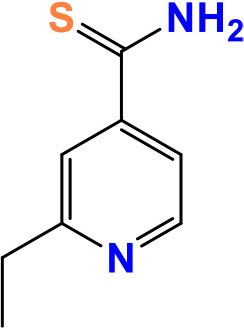 Pubchem CID: 2761171	Zhang et al., 1992; Vilchèze et al., 2006, 2008; Carette et al., 2011; Grant et al., 2016; Thee et al., 2016; Mori et al., 2017
*ethA* (*Rv3854c*)	1,470	489	55,326.1	Monooxygenase EthA	Activates the pro-drug ethionamide (ETH)			
*ethR* (Rv3855)	651	216	23,724.7	Transcriptional repressor protein EthR	Regulates negatively the production of ETHA			
KasA (Rv2245)	1,251	416	43,284.1	β -Ketoacyl acyl carrier protein synthase	Involved in fatty acid biosynthesis			
*thyA* (*Rv2764c*)	792	263	29,820.8	Thymidylate synthase ThyA	Deoxyribonucleotide biosynthesis	**PAS**: Inhibits of folic acid and thymine nucleotide metabolism **Activity**: Bacteriostatic	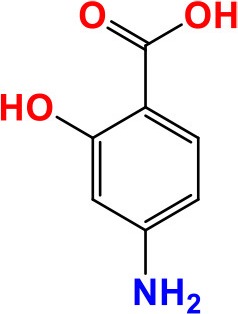 PubchemCID: 4649	Rengarajan et al., 2004; Mathys et al., 2009; Zhao et al., 2014; Meumann et al., 2015
*folC* (*Rv2447c*)	1,464	487	50,779.3	Folylpolyglutamate synthase protein FolC	Conversion of folates to polyglutamate derivatives			
*dfrA* (*Rv2763c*)	480	159	17,640	Dihydrofolate reductase DfrA	For de novo glycine and purine synthesis			
RibD (Rv2671)	777	258	27,693.5	Bifunctional enzyme riboflavin biosynthesis protein RibD	Involved in riboflavin biosynthesis			
*alr* (*Rv3423c*)	1,227	408	43,355.6	Alanine racemase Alr	Provides D-alanine required for cell wall biosynthesis	**DCS**: Inhibits the synthesis of peptidoglycan in the cell wall **Activity**: Bacteriostatic	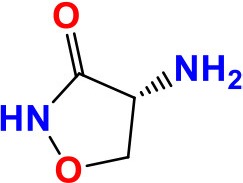 PubchemCID: 6234	Cáceres et al., 1997; Andini and Nash, 2006; Chen et al., 2012; Zhang and Yew, 2015; Desjardins et al., 2016
*ddl* (*Rv2981c*)	1,122	373	39,678.2	D-alanine: D-alanine ligase DdlA	Involved in cell wall formation			
Ald (Rv2780)	1,116	371	38,713.2	L-alanine dehydrogenase	Cell wall synthesis			
*cycA* (*Rv1704c*)	1,671	556	60,047.2	D-serine / alanine / glycine transporter protein CycA	transport across the cytoplasmic membrane			
Rv0678	498	165	18,346.7	Conserved protein	Transcription repressor for efflux pump MmpL5	**CLO**: Produces reactive oxygen, inhibits energy production, memberane distruption **Activity**: Bacteriostatic	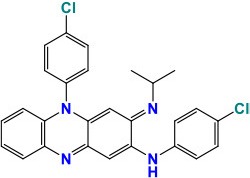 **Pubchem CID:** 2794	Huitric et al., 2010; Yano et al., 2011; Hartkoorn et al., 2014; Almeida et al., 2016
*Rv1979c*	1,446	481	51,084.1	Conserved permease	Probably involved in transportation of amino acid across the membrane.			
*pepQ* (*Rv2535c*)	1,119	372	38,758.9	Cytoplasmic peptidase PepQ	Possibly hydrolyses peptides			
*rplC* (Rv0701)	654	217	23,090.5	Encodes the 50S ribosomal L3 protein	Formation of peptidyltransferase center of the ribosome	**LIN/SZD**: Both act by fixation of an early step in protein synthesis **Activity**: Bactericidal	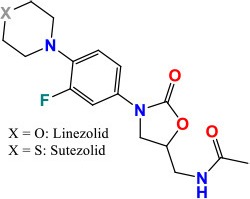 **Pubchem CID:** 441401	Williams et al., 2009; Beckert et al., 2012; Makafe et al., 2016; Zhang et al., 2016
*rrl* (MTB000020)	3,138	—–	—–	Ribosomal RNA 23S	Stable RNAs			
Ddn (Rv3547)	456	151	17,371	Deazaflavin-dependent nitroreductase	Converts bicyclic nitroimidazole drug candidate pa-824 to three metabolites,	**DMD**: Impeding the synthesis of mycolic acid **Activity**: Bactericidal	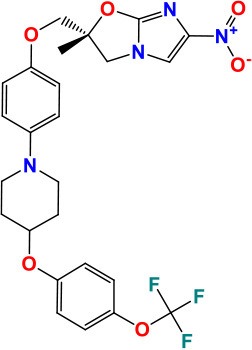 **Pubchem CID:** 6480466	Matsumoto et al., 2006; Palomino and Martin, 2014; Shimokawa et al., 2014; Bloemberg et al., 2015; Haver et al., 2015
Fgd1 (Rv0407)	1,011	336	490.783	F420-dependent glucose-6-phosphate dehydrogenase	Catalyzes oxidation of glucose-6-phosphate to 6-phosphogluconolactone using coenzyme F420 as the electron acceptor			
*fbiA* (Rv3261)	996	331	3,640.54	F420 biosynthesis protein FbiA	Required for coenzyme F420 production: involved in the conversion of FO into F420			
*fbiB* (Rv3262)	1,347	448	3,641.53	F420 biosynthesis protein FbiB	Required for coenzyme F420 production: involved in the conversion of FO into F420			
*fbiC* (Rv1173)	2,571	856	1,302.93	F420 biosynthesis protein FbiC	Participates in a portion of the F420 biosynthetic pathway between pyrimidinedione and FO			
*mmpL3* (*Rv0206c*)	2,835	944	100,872	Conserved transmembrane transport protein MmpL3	Unknown; Supposed to be involved in fatty acid transport	**SQ-109**: Acts by interfering with the assembly of mycolic acids into the bacterial cell wall core **Activity**: Bactericidal	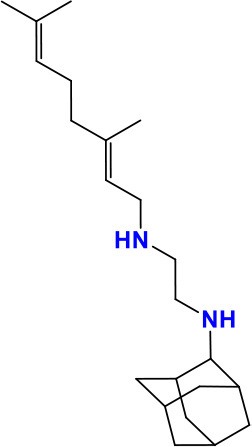 **Pubchem CID:** 5274428	Boshoff et al., 2004; Jia et al., 2005; Grzegorzewicz et al., 2012;
DprE1 (Rv3790)	1,386	461	50,163.2	Decaprenylphosphoryl-beta-D-ribose 2′-oxidase	Together with DPRE2 arabinan synthesis	**BTZ**: Drug is activated by reduction of an essential nitro group to a nitroso derivative, which can react with a cysteine residue in DprE1 **Activity**: Bactericidal	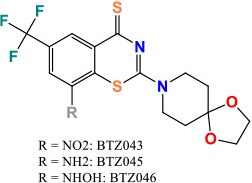 **ChemSpider ID:** 25941868	Manina et al., 2010; Trefzer et al., 2010; Kolly et al., 2014; Makarov et al., 2014
DprE2 (Rv3791)	765	254	27,468.8	Decaprenylphosphoryl-D-2-keto erythro pentose reductase	Together with DPRE1 arabinan synthesis			

### rpoB

RpoB is the target of RIF, a derivative of rifamycin. RpoB catalyzes the transcription of DNA into mRNA by using the four ribonucleoside triphosphates as substrates. In *M. tuberculosis* RIF binds with β-subunit of the RNA polymerase, encoded by *rpoB*, and inhibits the elongation of messenger RNA, thus interfering with transcription (Carlos and Martin, 2013; Piccaro et al., 2014). RpoB has a gene length of 3519 bp and conformational changes caused by mutations in *rpoB* can lead to RIF-resistance (Telenti et al., 1993). Ninety-six percent of RIF-resistance occurs within the “hot-spot region” (of 81 bp), also known as RIF-resistance determining region (RRDR), covering codons 507–533 of *rpoB* gene (Ramaswamy et al., 2003). Several studies have reported mutations in codons 516, 526 and 531 are most commonly found in RIF-resistant isolates (Ocheretina et al., 2014; Thirumurugan et al., 2015).

In particular, mutation in the *rpoB* gene at 531 codon (serine to leucine) is highly significant as it confers cross-resistance to rifabutin (Mboowa et al., 2014; Thirumurugan et al., 2015; Aye et al., 2016). whereas mutations at codons 516, 518, 526, and 529 are associated with low-level resistance to RIF and conserved susceptibility to other rifamycins, e.g., rifabutin or rifalazil (Cavusoglu et al., 2004; Tan et al., 2012). However, compensatory mutations were also identified in *rpoA* and *rpoC* encoding respectively for α and β' subunits of RNA polymerase (Comas et al., 2012). These compensatory mutations could be important for reinstating the fitness and emergence of MDR strains, and their transmissibility *in vivo* (Brandis and Hughes, 2013). Further studies are required to better understand their specific roles.

### *katG* and *inhA*

Mutations in *katG* and *inhA* are the main cause of resistance to INH in *M. tuberculosis*. KatG has a molecular mass of 80,572.8 Da and gene size of 2,223 bp while InhA has 28,527.8 Da molecular mass and 810 bp gene size, respectively. *katG* encodes multifunctional enzymes that exhibit both catalase-peroxidase and peroxynitritase activities, the former being important for activating the prodrug, and the latter in association with pathways involving reactive nitrogen and oxygen intermediates (Zhang et al., 1992). On the other hand, AhpC, SodC, KatG, and TpX are recognized as essential for virulence of the *M. tuberculosis* (Forrellad et al., 2013). NADH-dependent enoyl-acyl carrier protein (ACP)-reductase, encoded by *inhA*, is involved in the biosynthesis of mycolic acids and participates in the second reductive step in fatty acid biosynthesis (Rozwarski et al., 1998; Vilchèze et al., 2006). Mutational changes in these two genes, *katG* and *inhA*, are mainly associated with the resistance mechanism of INH (Ramaswamy et al., 2003).

Conversely, mutations in *Rv0340-0343, fadE24, efpA*, and *kasA* were identified in both INH-resistant and INH-susceptible strains, so their association with INH-resistance requires further clarification (Vilchèze et al., 2007). Among the *katG* mutations, S315T is considered as the most pervasive mutation that accounts for 40~94% resistance in MDR strains and results in reducing the ability of KatG to convert INH into isonicotinic acid, a precursor for the formation of INH-NAD adduct (Bantubani et al., 2014; Seifert et al., 2015; Aye et al., 2016). In addition, another newly reported *katG* mutation L101R identified in clinical isolates, changing an hydrophobic leucine to hydrophilic arginine, possibly involved in alteration of the conformation of binding protein near the active site and inhibited its bio-activation (Datta et al., 2016).

The second main cause of resistance to INH is due to mutations in the promoter region of *inhA*, resulting in the overexpression of *inhA*. The most frequently observed mutation in *inhA* regulatory region at position −15C/T is more generally associated with low level resistance to INH (MIC < 1 μg/ml) (Banerjee et al., 1994; Fenner et al., 2012; Aye et al., 2016). Moreover, double mutations at −8T/C, −15/T (Zhang and Yew, 2015), and −17C/T (Müller et al., 2011) in the *inhA* promoter region were also found to be associated with INH-resistance. Some recent studies have described that a mutation in the regulatory region of *inhA*, together with a mutation in *inhA* coding region, results in high-level resistance against INH as well as cross-resistance against the structurally related ethionamide (Machado et al., 2013).

### *embB* and *embC*

EmbB and EmbC have molecular masses of 118,021 Da, and 117,490 Da, respectively, and gene sizes of 3,297 bp, 3,285 bp, respectively. Mutations in *embB* and *embC* cause resistance against ethambutol [EMB; dextro-2,2′-(ethylenediimino)-DI-1-butanol], through restricting the action of drug to cease the biosynthesis of mycobacterial cell wall. In *M. tuberculosis*, the genes *embCAB*, are organized as an operon that encodes arabinosyl transferases, involved in the synthesis of arabinogalactan. (Mikusová et al., 1995). EMB plays intrusive role in biosynthesis of arabinogalactan in the cell wall to convert multiplying bacilli into a bacteriostatic phase (Wang et al., 2010). Mutations in the *embCAB* operon were described to cause alterations in the drug-protein binding site (Xu et al., 2015). Numerous studies concluded that a mutation at codon306 in *embB* was the cause of EMB-resistance (Shi et al., 2011a; Yoon et al., 2013; Moure et al., 2014).

It was also reported that mutations in genes of the decaprenylphosphoryl-beta-D-arabinose (DPA) biosynthesis and utilization pathway genes, *Rv3806c* and *Rv3792*, combined with mutations in *embB* and *embC* genes increased the MICs ranges of EMB, depending on mutation type and number (Shi et al., 2011a; Safi et al., 2013). A strong association was also noticed between the Met306Val and Met306Leu at *emb306* and EMB-resistance (Zhang et al., 2014), which suggests the usefulness of the *embB*306 mutation to serve as potential molecular markers for EMB resistance. However, in 30% EMB-resistant *M. tuberculosis* strains there was no mutation in *embB*, alluding to the need to intensively search for other mechanisms of EMB resistance (Shen et al., 2007; Perdigão et al., 2009). Interestingly, in another study, 70% mutations were found in 306, 406 or 497 codons, 13% mutations outside the three regions between codons 296 and 426 and 15% mutations in the *embC-embA* intergenic region among the total 98% of mutations in the *embCAB* locus (Brossier et al., 2015). Recently, mutations in *embB* along with mutations in *ubiA*, encoding for a decaprenylphosphoryl-5-phosphoribose (DPPR) synthase associated with arabinogalactan synthesis pathway, were associated with high level of resistance to EMB (Tye et al., 2015).

### *pncA, rpsA, panD, clpC1*, and *Rv2783c*

Pyrazinamide (PZA) is an important front-line anti-TB drug. In *M. tuberculosis*, resistance to PZA is mainly associated with *pncA, rpsA*, and *panD*. However, some other proteins such as *ppsA* encoding polyketide synthase involved in phthiocerol dimycocerosate (PDIM) synthesis, cell division protein FtsH, TetR family transcriptional regulator (3R)-hydroxyacyl-ACP dehydratase subunit HadC, phosphate ABC transporter permease protein PstC2 and transmembrane transport protein MmpL4 found to be responsible for PZA resistance (Zhang et al., 2017). Pyrazinamidase (Pzase), encoded by the 561-bp *pncA*, converts the pro-drug PZA into pyrazinoic acid (POA, its active form). PZA resistance in *M. tuberculosis* is most commonly associated with mutations in the whole open reading frame or 82–262 bp regulatory region of *pncA* gene (Juréen et al., 2008).

POA interrupts the bacterial membrane activities. POA and its n-propyl ester may have the ability to constrain the fatty acid synthase type I in replicating *M. tuberculosis* bacilli, ineffective efflux pump also may result in accumulation of protonated POA during acidic conditions resulting in bacterial cell damage (Scorpio and Zhang, 1996; Zimhony et al., 2007; Shi et al., 2011b, 2014; Njire et al., 2016). The 481-amino acid-long 30S ribosomal protein S1 is encoded by *rpsA*. It plays a significant role in mRNA translation (involving a short shine-dalgarno (SD) purine-rich sequence), and in trans-translation, a unique process that utilizes transfer-messenger RNA (tmRNA) molecule to rescue stalled ribosomes. POA was also associated with disruption of the trans-translation process through binding to the 30S ribosomal protein S1 (RpsA) (Shi et al., 2011b). In the work of Shi et al. (2011b), deletion of alanine, resulting from a GCC deletion at 438 bp (C-terminus) of *rpsA*, was found in PZA-resistant strains lacking mutations in *pncA* gene. Such mutation was thought to induce resistance against POA and, accordingly, increased level of resistance were observed after overexpression of *rpsA* (Shi et al., 2011b).

Later, many studies presented disparate results about the mutations in *rpsA* in both PZA-resistant and PZA-susceptible isolates (Bhuju et al., 2013; Maslov et al., 2015). However, only synonymous mutations were reported in *rpsA* (A636C and G960A) for both PZA-resistant and PZA-susceptible clinical strains (Alexander et al., 2012). In another study, mutations in *rpsA* gene were found in 3/52 PZA-resistant and 1/108 PZA-susceptible clinical isolates (Tan et al., 2014). This absence of clinical isolates presenting *rpsA* mutations may indicate a strong fitness cost associated with such mutations, although more investigations are required to fully understand the link between RpsA and the activity of PZA. The third gene *panD*, of 420 bp in size, is putatively involved in pantothenate biosynthesis through converting L-aspartate into beta-alanine (Shi et al., 2014). Some studies found mutations in *panD* gene of PZA-resistant isolates lacking mutations in *pncA* and *rpsA* (Pandey et al., 2016).

Among the three genes identified so far as potentially linked with resistance to PZA, i.e., *pncA, rpsA*, and *panD*, the most frequent mutations (>80%) were found to occur in the *pncA* gene (Xia et al., 2015; Xu et al., 2016). Recently, a new target of PZA *clpC1* (*Rv3596c*) was identified, encodes an ATP-dependent ATPase which is involved in protein degradation by forming a complex with protease ClpP1 and ClpP2 (Zhang et al., 2017). ClpC1 is an 848-amino acids containing protein and the mutation (G296T) change amino acid G99D cause resistance to PZA. Interestingly, mutations in *clpC1* has also been reported as a resistance factor against three new cyclic peptide antibiotics including cyclomarin A (Schmitt et al., 2011), lassomycin at the N-terminal repeat region at Q17R, R21S, and P79T (Gavrish et al., 2014) and ecumicin at L92S or F or L96P sites (Gao et al., 2015). The three new drug candidates appear to bind at the different sites of the ClpC1. The gene *Rv2783c* encoding a bifunctional enzyme was indicated as a new target very recently (Njire et al., 2017). Rv2783 was proved to be able not only to catalyze metabolism of RNA and single-stranded DNA, but also to metabolize ppGpp, an important signal transducer involved in the stringent response in bacteria.

### *rpsL, rrs, gidB, eis*, and *tlyA*

The *rpsL* gene is very small, only 375-bp, while *rrs* gene is much larger, with 1,537 bp but both are associated with resistance to STR. The *rpsL* encodes 30S ribosomal protein S12 RpsL involved in the initiation step of RNA translation, while *rrs* encodes 16S rRNA. Mutations in *rpsL* and *rrs* are known to cause resistance against STR (Finken et al., 1993), the first anti-TB drug discovered. STR is an aminoglycoside and targets the actively growing bacteria by hindering the translation of protein synthesis (Chakraborty et al., 2013). More precisely, STR interacts with formyl-methionyl-tRNA to bind the 30S subunit of the ribosome at the ribosomal protein S12 encoded by *rpsL* gene and also at 16S rRNA encoded by *rrs* gene, preventing normal functioning of the ribosome (Sharma et al., 2007).

The most commonly reported mutation in *rpsL* is the replacement of lysine into arginine at positions 43 and 88. Regarding *rrs*, mutations around nucleotides 530–915 make *M. tuberculosis* strains highly resistant to STR (Jagielski et al., 2014; Zhao et al., 2014). Additionally, a 675 bp gene, *gidB*, encoding for a conserved 7-methylguanosine methyltransferase specific for the 16S rRNA, was found to confer intermediate-level of resistance toward STR by conferring the A80P mutation to the gene product GidB (Perdigão et al., 2014). Generally, mutations in the *rpsL* gene covers around 80% of STR-resistance (Jnawali et al., 2013). More precisely, the mutations at codons 43 and 88 in *rpsL* gene were identified in more than 60% of STR-resistance cases, while 17% contained *rrs* mutations and 14% were observed with mutations in *gidB*, in a panel of 161 clinical isolates (Smittipat et al., 2016).

Some STR-resistant *M. tuberculosis* strains also conferred cross-resistance with kanamycin (KAN) and amikacin (AMK). The mutation at the codon A1401G in the *rrs* gene in particular was associated with high-level resistance to KAN and AMK, along with cross-resistance to capreomycin (CAP). Whereas mutations C1402T or G1484T were also associated with CAP resistance in addition cross-resistance with KAN or viomycin (VIM) (Du et al., 2013; Sowajassatakul et al., 2014). Similarly, mutations at position 1,400, 1,401, and 1,483 bp in the *rrs* gene were linked with high-level resistance to both KAN and AMK in KAN-resistant strains (Ajbani et al., 2011; Yuan et al., 2012). Moreover, mutations in the promoter region of the *whiB7* gene, with a role in transcriptional mechanisms, were shown to be associated with resistance to an array of antibiotics, and increased expression of the efflux enhanced by *atpE* and the *eis* promoter region was also observed (Reeves et al., 2013).

Further, *eis* and *tlyA* (1,209 bp and 807 bp in size, respectively) were also found to have putative roles in resistance. *eis* encodes an aminoglycoside acetyltransferase and mutation at position −10 and −35 of the *eis* promoter has been associated with the low-level resistance to KAN (Ajbani et al., 2011; Yuan et al., 2012; Du et al., 2013; Reeves et al., 2013; Sowajassatakul et al., 2014). *tlyA* encodes rRNA methyltransferase, specific for 2′-O-methylation of ribose in rRNA. Expression of *tlyA* gene (commonly A1408G) affects both drug susceptibility and fitness cost of drug resistance through methylation mechanisms (Freihofer et al., 2016). However, another study did not find any mutation in the *tlyA* gene (Du et al., 2013). Besides, overexpression of Rv0148 protein play a vital role in three-fold increase in MIC of AMK and two-fold of KAN (Sharma et al., 2015). While overexpression of ferritin (*Rv3841*/*bfrB*) showed an alternate behavior, there was two-fold increase in MIC of AMK and three-fold of KAN (Sharma et al., 2016). Regarding to above discussed genes and their role in development of resistance, it is important to note that KAN and AMK are aminoglycosides and CAP and VIM are cyclic peptide antibiotics, they have similar structures and bind on the same location in the ribosome, at the interface of the small and large subunits (Stanley et al., 2010). The similarity of the mechanisms of actions and their resistance development simplifies understanding to some extent.

### *gyrA* and *gyrB*

*gyrA* and *gyrB* are the two main genes with mutations linked to resistance against fluoroquinolones (FQ's). *gyrA* is 2517-bp long while *gyrB* gene is 2,028-bp. Mutations in the quinolone resistance-determining region (QRDR) of *gyrA* (74–113 codons) and *gyrB* (codon 500–540) in *M. tuberculosis* are largely responsible for generating the resistance to FQ's (Pantel et al., 2012; Nosova et al., 2013). Two subunits α and β each of *gyrA* and *gyrB*, that encode topoisomerase II (DNA gyrase) catalyzing the supercoiling of DNA, furnishes the most important target of FQ action (Aubry et al., 2004). The most commonly found mutations at position Ala-74, Gly-88 Ala-90, Ser-91, and Asp-94 of *gyrA* generally result in high-level resistance against FQ (Li et al., 2014).

A natural polymorphism at position 95 (Ser or Thr) in *gyrA* is not related to FQ's-resistance since it is also found in FQ-sensitive strains (Nosova et al., 2013). Usually, mutations in *gyrB* (10–15%), being less commonly found among clinical isolates, induce low-level resistance to FQ's (Cui et al., 2011). However, the synchronized mutations in both *gyrA* and *gyrB*, such as Ala543Val (*gyrB*)-Asp94Asn/Asp94Gly (*gyrA*) and Asn538lle (*gyrB*)-Asp94Ala (*gyrA*) persuaded very high resistance against FQ's (Long et al., 2012). An interested study identified that presence of mutations T80A and A90G in *gyrA* led to hypersusceptibility to several quinolones (Aubry et al., 2006). Similarly, most of the mutations in the *gyrA* Asn538Asp and Asp500His in *gyrB* are shown to be associated with cross-resistance among the FQs, whereas in *gyrB* the mutation Arg485His does not confer any resistance (Nosova et al., 2013). One intriguing study found a *M. tuberculosis* strain with Asn533Thr mutation in *gyrB* showing susceptibility to ofloxacin (OFX) but resistance to moxifloxacin (MOX) and gatifloxacin (Von Groll et al., 2009).

Proteomic comparative study enlightened the 11 proteins (Rv1080c, Rv1827, Rv2623, Rv1636, Rv0952, Rv1932, Rv0009, Rv0054, Rv2889c, Rv3418c, and Rv3914) that were overexpressed in the presence of OFX and MOX. Among them, Rv1636, Rv2623, and Rv1827 were reported with unidentified functions. Molecular docking and InterProScan investigation explored the interaction of conserved domain of hypothetical proteins with these both drugs which possibly directs the inhibition of functions of these proteins that might be overexpressed to repress this effect (Lata et al., 2015b).

In another study, the same author reported 14 proteins with more intensities in OFX resistant isolates as compare to susceptible. Interaction of conserved domains and motifs of hypothetical proteins (Rv3551, Rv2744c, Rv0148, and Rv2140c) and OFX was revealed through molecular docking, these findings indicated the role of these proteins in resistance mechanisms as well as prospective drug targets (Lata et al., 2015a). Drug efflux mechanisms or changes in the membrane permeability to agents might constitute alternative resistance mechanisms regarding FQ resistance in *M. tuberculosis* (Escribano et al., 2007). A study corroborating such ideas has found that 30% of FQ's-R strains do not harbor any mutation in the QRDRs of both *gyrA* and *gyrB* genes (Alvarez et al., 2014). In a recent study by Pucci et al., *M. tuberculosis* GyrB inhibitors (SPR-720, SPR-750) have proved to be attractive for developing novel drugs against TB without cross-resistance to known quinolones and showing very good activity *in vivo*.

### *ethA* and *ethR*

*ethA* and *ethR* are associated with resistance to ethionamide (ETH) (2-ethylpyridine-4-carbothioamide) and prothionamide (2-propylpyridine-4-carbothioamide) which are important drugs for the treatment of MDR-TB and TB meningitis in both adults and children (Thee et al., 2016). The 1,470-bp long *ethA* encodes the monooxygenase EthA which bio-activates the pro-drug ETH into its active form (Grant et al., 2016). EthA is negatively regulated by the transcriptional repressor EthR (Carette et al., 2011). ETH is derived from nicotinic acid having a similar structure to INH. The action of ETH is to interrupt mycolic acid synthesis by establishing an adduct with NAD that inhibits the enoyl-ACP reductase InhA (Mori et al., 2017). Mutations in *ethA*/*ethR*, as well as mutations in *inhA* or its promoter cause the resistance to both INH and ETH (Carette et al., 2011). Accordingly, −15C to −15T mutation in the promoter region of *inhA* and S94A (serine to alanine) and I194T (isoleucine to threonine) mutations in the gene product InhA were found to confer high-level resistance against INH and ETH in MDR-TB (Machado et al., 2013). Furthermore*, mshA*, a gene encoding a glycosyltransferase involved in mycothiol biosynthesis, has also been considered as a possible resistance target for ETH (Vilchèze et al., 2008).

### *thyA* and *folC*

The 792-bp long *thyA*, encoding thymidylate synthase A, has an important role in deoxyribonucleotide biosynthesis, by providing the sole *de novo* source of dTMP through conversion of deoxyuridine monophosphate (dUMP) to deoxythymidine monophosphate (dTMP). Interacting with folate metabolism is essential, especially involving tetrahydrofolate as a “methyl” donor. In an interesting study, mutations in *thyA* gene were identified by transposon mutagenesis were associated with resistance to PAS (Rengarajan et al., 2004). Some studies have affirmed Thr202Ala in *thyA* as the most common mutation associated with PAS-resistance, though a few susceptible isolates containing the same mutation have also been observed (Meumann et al., 2015). According to recent studies, the 1464-bp long *folC* gene (encoding FolC-dihydrofolate synthase/ folylpolyglutamate synthase), with various missense mutations were found to confer resistance to PAS in laboratory isolates of *M. tuberculosis*. The main function of FolC, the bifunctional enzyme, is to convert folates into polyglutamate derivatives, in simplistic terms. Bacteria require folate for the biosynthesis of glycine, methionine, formyl-met-tRNA, thymidylates, purines and pantothenate. In 85 MDR-TB clinical isolates, mutations in *folC* were identified in five PAS-resistant isolates (Zhao et al., 2014). In another study, only 37% of PAS-resistant strains had mutations in *thyA*, suggesting that other mechanisms of resistance to PAS would need to be explored (Mathys et al., 2009).

### *rplC* and *rrl*

*rplC* and *rrl* genes have been associated with different levels of phenotypic resistance to Linezolid (LZD) and Sutezolid (PNU-100480, SZD) in *M. tuberculosis* (Makafe et al., 2016; Zhang et al., 2016). *rplC* gene has 654 bp in length and encodes the 50S ribosomal L3 protein, and may contribute in the synthesis of the ribosomal peptidyltransferase. Whereas, *rrl* is 3138 bp long and encodes 23S ribosomal RNA. LZD and SZD belonging to the oxazolidinone class, act by fixation of an early step in protein synthesis, through binding of the assembly initiator protein directly near the 3′-end of 23S rRNA, where nucleation of the assembly of the 50S subunit is performed (Williams et al., 2009). However, recently, the mutation T460C in *rplC* of LZD-resistant strain (Cys154Arg) has been repeatedly shown to be of considerable importance (Williams et al., 2009; Beckert et al., 2012; Makafe et al., 2016; Zhang et al., 2016). Moreover, *in vitro* selected LZD-resistant mutants with G2576T and G2061T mutations in *rrl* gene had MICs of 16–32 μg/ml, while susceptible strains without mutations showed MICs of 4–8 μg/ml (Hillemann et al., 2008; Zhang et al., 2016). The mutations in these two genes are associated with only 29.4% of LZD-resistance in *M. tuberculosis* (Islam et al., 2017). So, further resistance mechanisms need to be explored.

### *Rv0678* and *atpE*

The 498-bp *Rv0678* encodes a conserved protein with a molecular mass of 18,346.7 (Da). Some recent studies recommended that mutations in the transcriptional regulator of Rv0678 up-regulated MmpL5, a multi-substrate efflux pump, causing resistance not only to CLO (a riminophenazine compound) but also to BDQ, a diarylquinoline (Andries et al., 2014). Some studies suggested the outer membrane of *M. tuberculosis* as the possible target of CLO (Yano et al., 2011). The main factor for resistance to CLO other than mutations in the transcriptional regulator of Rv0678 is not yet fully characterized (Hartkoorn et al., 2014). The *atpE* gene is known to encode the subunit C of the ATP synthase, AtpE (lipid-binding protein) (dicyclohexylcarbodiimide-binding protein), a complex structure that generates the ATP needed by the mycobacterial cell.

Mutations in the 246-bp long *atpE* gene (*Rv1305*) are thought to be related to the resistance to BDQ with the most frequently detected mutations including A63P and I66M (Andries et al., 2005; Koul et al., 2007). BDQ has a favored specificity toward the mycobacterial ATP synthase, as compared to mitochondrial ATP synthase in exerting its therapeutic action (Haagsma et al., 2009), but the mutations in AtpE (A63P and I66M) disturbs such inhibition against bacterial ATP synthase C. However, out of 53 strains of *M. tuberculosis* only 15 displayed mutations while 38 strains lacked mutations in *atpE* or even in the F0, F1 operons (Huitric et al., 2010), which strongly implicates that some other resistance mechanisms against BDQ might be present. Recently, mutations in *pepQ* (*Rv2535c*, a putative Xaa-Pro aminopeptidase) were proposed to be able to confer cross-resistance between BDQ and CLO. Whereas mutations in *Rv0678* and *pepQ* were noticed at a similar rate in a wild-type population treated with BDQ or CLO in mice. Mutations in *Rv0678* and *pepQ* regulate the significant mechanisms of clinical resistance to BDQ and CFZ (Almeida et al., 2016).

### *alrA, cycA*, and *emr37*

*alrA* encodes D-alanine racemase AlrA, required for the conversion of L-alanine to D-alanine (Palomino and Martin, 2014). D-alanine ligase inhibits the production of peptidoglycan conductive to cell wall formation in bacteria. D-cycloserine (DCS) is a time-honored oral bacteriostatic anti-TB drug used in treatment of TB, especially MDR-TB and XDR-TB (Zhang and Yew, 2015). The absolute target of DCS in *M. tuberculosis* is still not fully identified. However, according to some prior studies, overexpression of AlrA triggered resistance to DCS in recombinant mutants of *Mycobacterium smegmatis* (Cáceres et al., 1997). Interestingly, a very recent study reported that loss-of-function mutations in *ald* (*Rv2780*), encoding L-alanine dehydrogenase, were also associated with resistance to DCS (Desjardins et al., 2016).

Another gene *cycA*, of 1,671 bp in size, encodes a D-alanine transporter. The point mutation in *cycA* has been considered a possible cause for resistance in *M. bovis* against DCS (Chen et al., 2012). Low cell wall permeability and the expression of *emr37*, a gene that collates methylase at a specific site in the 23S rRNA, hindering the binding of the antibiotic has been ascribed as the key factor of resistance to macrolides leading four to eight-fold rise in MIC values (Andini and Nash, 2006).

### *ddn, fgd1*, and *fbia/B/C*

*ddn* has a gene size of 456 bp and encodes deazaflavin-dependent nitroreductase, while *fgd1* with a size of 1,011 bp encodes F420-dependent glucose-6-phosphate dehydrogenase Fgd1. *fbiA/B/C* is a complex/operon of three genes with 996, 1,347, and 2,571 bp gene sizes, respectively. These three genes are mainly required for coenzyme F420 production for the biosynthesis of protein FbiA, FbiB, and FbiC sequentially. *ddn, fgd1*, and *fbiA/B/C* gene products were proposed as the targets of DMD and pretomanid (PA-824, PRM). DMD is a derivative of nitro-dihydro-imidazooxazole and acts by impeding the synthesis of mycolic acid in a distinct fashion compared to INH, as it only inhibits methoxy- and keto-mycolic acid synthesis while INH also inhibits α-mycolic acid (Matsumoto et al., 2006; Palomino and Martin, 2014). Mutations in *ddn, fgd1*, and *fbiA/B/C* have been proposed to be a mechanism of resistance to DMD and PRM in *M. tuberculosis* (Shimokawa et al., 2014).

A very recent study reported mutations in the *fbiA* and *fgd1* genes associated with *M. tuberculosis* resistance to DMD in a patient with formidable drug-resistant TB (Bloemberg et al., 2015). PRM is a nitroimidazole derivative with activity against both latent TB and active TB as tested under aerobic and anaerobic conditions (Stover et al., 2000). PRM is a prodrug that needs a metabolic activation principally by a deazaflavin (cofactor F420)-dependent nitroreductase (Ddn) (Manjunatha et al., 2006). It also acts by inhibiting mycolic acid synthesis. A very recent assay of drug metabolism genes found potential resistance to PRM, and detected mutations frequency higher than those previously reported (Stover et al., 2000; Haver et al., 2015). However, further studies are essential to better delineate the mechanisms underlying bacillary resistance.

### mmpl3

Mutations in *mmpL3* were attributed to resistance in *M. tuberculosi*s against SQ-109 (1,2-ethylenediamine), a synthetic analog of EMB (Tahlan et al., 2012). The 2,835-bp MmpL3 is a transmembrane transport protein presumably involved in fatty acid transport. SQ109 acts by interfering with the assembly of mycolic acids into the bacterial cell wall, resulting in accumulation of trehalose monomycolate, a precursor of the trehalose dimycolate. Similarly other cell wall inhibitors such as INH and EMB, SQ-109 induces the transcription of *iniBAC* operon required for efflux pump functioning (Boshoff et al., 2004). Its synergistic effects with BDQ *in vitro* and interactions with SZD were also observed. Additionally, mutations in the *mmpL3* gene of spontaneously generated SQ-109-resistant mutants has suggested *mmpL3* as the target of SQ-109 by virtue of the transporter mechanism discussed above (Grzegorzewicz et al., 2012). In *M. tuberculosis* strains resistant to INH, EMB and SQ109, there is an up-regulation of *ahpC*, signifying a possible role of this gene in the development of resistance against these drug(s) (Jia et al., 2005).

### *dprE1* and *dprE2*

*dprE1* (*Rv3790*) and *dprE2* (*Rv3791*) encode decaprenylphosphoryl-beta-D-ribose 2′-oxidase and decaprenylphosphoryl-D-2-keto-beta-erythro-pentose reductase, respectively. These genes encode proteins that catalyze the two-step epimerization of decaprenylphosphoryl ribose (DPR) to decaprenylphosphoryl arabinose (DPA) in the arabinan synthesis pathway, which is essential for building the bacterial cell wall (Kolly et al., 2014). *dprE1* and *dprE2* were the possible targets of 1,3-benzothiazin-4-one or benzothiazinone (BTZ) (Makarov et al., 2014). BTZ is activated in the bacteria by reduction of an essential nitro group to a nitroso derivative, which can react with a key cysteine residue in DprE1 and form a covalent adduct (Trefzer et al., 2010). In spontaneous BTZ-resistant mutants, amino-acid Cys387 of DprE1 was replaced by Ser or Gly. On the other hand, in *M. avium*, which is naturally resistant to BTZ, amino-acid Cys387 was replaced by an Ala. An alternative BTZ-resistance mechanism has recently been described in *M. smegmatis*. Overexpression of nitroreductase NfnB indeed inactivated the drug by reducing its critical nitro-group into an amino-group. This was facilitated by a common amino acid stretch between NfnB and DprE1 (Manina et al., 2010). *M. tuberculosis*, however, seems to lack nitroreductases which enables it to inactivate this drug. Nevertheless, this finding could be important for development of new BTZ analogs with improved activity.

## Novel therapeutic drug targets and molecular mechanisms

There are some new findings about therapeutic drug targets of new anti-TB drugs. Very recently, VCC234718, a molecule found to be very active against *M. tuberculosis* with growth inhibition activity. In a VCC234718-resistant mutant, a Y487C resistance-conferring substitution was identified in the inosine monophosphate dehydrogenase, GuaB2, which was subsequently recognized to be the prime molecular target of VCC234718 (Singh et al., 2017). Similarly, another study reported a potential promoter mutation in upstream of *guaB2* in a non-cytotoxic indazole sulfonamide resistant mutant (Park et al., 2017).

Conversely, 11 eukaryotic-like serine/threonine protein kinases (STPKs) were observed in *M. tuberculosis*, which are considered as key components in cell growth, signal transduction and pathogenesis. An interesting study discovered the binding proteins in *M. tuberculosis* for all of the STPKs, and constructed the first STPK protein interaction (KPI) map that includes 492 binding proteins and 1,027 interactions. Functional investigations confirmed that PknG regulates cell wall integrity through peptidoglycan (PG) biosynthesis, e.g., MurC (Wu et al., 2017). A better understanding of biochemical pathways in *M. tuberculosis* is essential for the development of new and efficient chemotherapeutics.

Two important enzymes, isocitrate lyase (ICL) and malate synthase (MS) play vital role in regulation of glyoxylate shunt, a pathway required by *M. tuberculosis* to metabolize fatty acids (FAs). ICL may facilitates in survival of *M. tuberculosis* during the acute or chronic phases of infection through physiologic activities apart from fatty acid metabolism. MS mediates growth and survival on fatty acids through its potential ability to simultaneously detoxify a metabolic byproduct arising from the initial assimilation of acetyl coenzyme A (acetyl-CoA), glyoxylate, while assimilating a second molecule of acetyl-CoA. Depletion of MS during acute or chronic phase infections kills TB bacilli. These studies recommend MS as a potential drug target in *M. tuberculosis* (Puckett et al., 2017).

The cytoplasmic phases in peptidoglycan biosynthetic pathway are catalyzed by the Mur (A-F) enzymes. The imperative role of Mur enzymes in cell integrity and the minimum availability of their complements in eukaryotes highlighted them as promising anti-TB drug targets (Eniyan et al., 2016). Besides these, several other potential targets have also been proposed, such as, MbtA—involved in the iron metabolism of *M. tuberculosis*; cytochrome b subunit (QcrB) and type II NADH dehydrogenase—involved in energy generation; fatty acid synthases (FASs) and polyketide synthases (PKSs) involved in cell wall biosynthesis (Fernandes et al., 2015). Phenyl-diketo acid (PDKA) predicted to be the inhibitor for malate synthase (*glcB*) that characterize fatty acid metabolism (Krieger et al., 2012). Cyclospropane synthase (*pcaA*) involved in mycolic acid synthesis, presumed to be the significant target of sinefungin, thiacetazone, s-adenosyl-N-decylaminoethyl (Vaubourgeix et al., 2009). Furthermore, polyketide synthase (*pks13*) and acyl-AMP ligase (*fadD32*) are also responsible in mycolic acid synthesis, however, they are considered as the imperative targets of Cerulenin; Thiopene and 4,6-diaryl-5,7-dimethyl coumarin, respectively (Gavalda et al., 2009; Stanley et al., 2013; Wilson et al., 2013). Propanamide [5-methyl-N-(4-nitrophenyl) thiophene-2- carboxamide and 3-phenyl-N-(4-piperidin-1- ylphenyl) carbamothioyl] was approved as an efficient inhibitor against CTP synthase (*pyrG*) pyrimidine which is responsible to regulate metabolic processes (Mori et al., 2015).

Similarly, in another study Rhodanine analogs provided promising results by inhibiting mAGP complex synthesis directing through dTDP-keto-deoxyglucose epimerase (*rmlC*) (Ren et al., 2015). 4-diphosphocytidyl-2-Cmethylerythritol synthetase (*ispD*) involved in isopentenyl diphosphate biosynthesis assumed to be inhibited by Domiphen bromide (Gao et al., 2012). Consequently, shortening the TB therapy, simplifying DS-TB as well as DR-TB regimens and increasing the cure rates are the prime goals of newly developed or repurposed compounds (Brigden et al., 2014).

## Concluding remarks

In conclusion, DR-TB (MDR, XDR, and TDR) is an intensifying health crisis around the globe, mainly distressing economically active young adults and responsible for high mortality rate regardless of HIV status (Dheda et al., 2014). The genetic modifications underlying the profound drug resistance of *M. tuberculosis* immensely require insight investigations to unravel the complex pathways which hamper not only the clinical application of available drugs but also the development of new anti-TB drugs. The frequency of drug resistance continues toward upset level when TB regimens are practiced without fully exploration of resistance mechanisms. Likewise, the absence of effective drugs is the prime reason for rapid emergence of DR-TB. Hopefully the new knowledge along with vigilant understanding of alterations in genomics of drug targets and drug resistance mechanisms in *M. tuberculosis* would be tremendously helpful in development of new and highly effective anti-TB drugs.

## Author contributions

HH, MI, CC, CW, and TZ: Conceived and designed the initial draft to discuss the issues; HH, MM, and TZ: Wrote the manuscript; HH, YL, YT, XL, ST, and TZ: Assisted in evaluation of the article; WY, VD, HH, JL, and TZ: Critically assessed and guided up to final version. All the authors contributed to highlight related concerns and approved the final version.

### Conflict of interest statement

The authors declare that the research was conducted in the absence of any commercial or financial relationships that could be construed as a potential conflict of interest.

## Abbreviations

TB: Tuberculosis
*M. tuberculosis*: *Mycobacterium tuberculosis*
BCG: Bacille-Calmette-Guérin
*M. smegmatis*: *Mycobacterium smegmatis*
*M. bovis*: *Mycobacterium bovis*
*M. avium*: *Mycobacterium avium*
FLD's: First line drugs
SLD's: Second line drugs
STR: Streptomycin
INH: Isoniazid
PZA: Pyrazinamide
RIF: Rifampicin
FQ: Fluoroquinolone
AMK: Amikacin
KAN: Kanamycin
CAP: Capreomycin
RFB: Rifabutin
CLO: Clofazimine
CLR: Clarithromycin
THZ: Thiacetazone
BDQ: Bedaquiline
DMD: Delamanid
EMB: Ethambutol
POA: Pyrazinoic acid
VIM: Viomycin
LZD: Linezolid
SZD: Sutezolid
DCS: D-cycloserine
PRM: Pretomanid
BTZ: Benzothiazinone
ETH: Ethionamide
PAS: Para-aminosalicylic acid
MDR-TB: Multidrug resistance tuberculosis
XDR-TB: Extensively drug resistance tuberculosis
TDR-TB: Totally drug resistance tuberculosis
HIV: Human-immunodeficiency virus
WHO: World health organization
MIC: Minimum inhibitory concentration
TEM: Transmission electron microscopy
AFM: Atomic force microscopy
MGIT96: Mycobacteria Growth Indicator Tube
LPA: Line probe assay
MODS: Microscopic observation drug susceptibility assay
AFB: Acid-fast bacilli
SNP: Single nucleotide polymorphisms
ETL: Electron-transparent layer
EOL: Outer electron-opaque layer
TNF-α: Tumour necrosis factor alpha
RRDR: RIF-resistance determining region
ACP: Enoyl-acyl carrier protein
DPPR: Decaprenylphosphoryl-5-phosphoribose
PDIM: Phthiocerol dimycocerosate
QRDR: Quinolone resistance determining region
dUMP: deoxyuridine monophosphate
dTMP: deoxythymidine monophosphate.
